# Complex Response of White Pines to Past Environmental Variability Increases Understanding of Future Vulnerability

**DOI:** 10.1371/journal.pone.0124439

**Published:** 2015-04-17

**Authors:** Virginia Iglesias, Teresa R. Krause, Cathy Whitlock

**Affiliations:** 1 Montana Institute on Ecosystems, Montana State University, Bozeman, Montana, United States of America; 2 Department of Earth Sciences, Montana State University, Bozeman, Montana, United States of America; Ecole Pratique des Hautes Etudes, FRANCE

## Abstract

Ecological niche models predict plant responses to climate change by circumscribing species distributions within a multivariate environmental framework. Most projections based on modern bioclimatic correlations imply that high-elevation species are likely to be extirpated from their current ranges as a result of rising growing-season temperatures in the coming decades. Paleoecological data spanning the last 15,000 years from the Greater Yellowstone region describe the response of vegetation to past climate variability and suggest that white pines, a taxon of special concern in the region, have been surprisingly resilient to high summer temperature and fire activity in the past. Moreover, the fossil record suggests that winter conditions and biotic interactions have been critical limiting variables for high-elevation conifers in the past and will likely be so in the future. This long-term perspective offers insights on species responses to a broader range of climate and associated ecosystem changes than can be observed at present and should be part of resource management and conservation planning for the future.

## Introduction

Climate change projections in the western United States include temperature increases of 3–5°C [1], decreased winter snowpack, and increased moisture deficits in the coming decades [2]. The magnitude of these changes will stress ecosystems, particularly those near their climate limits. Future scenarios for northern Rocky Mountain conifer forests include dramatic shifts in species biogeographic ranges, loss of high-elevation conifers, changes in community composition, and greater risk of fire and insect infestations [3]. Understanding how individual species will respond to this suite of future climate and biotic pressures has become an important goal in the conservation and management of protected lands.

The concept of an ecological niche (i.e., n-dimensional space defined by the environmental conditions that comprise the physiological requirements of an individual, population, or species [4]) is a guiding approach for estimating past and future probability of species presence in geographic space. Ecological niche modeling defines species distributions based on mathematical representations of the known biogeographic range (i.e., realized ecological niche), most commonly defined as a function of present-day or recent historical climate. Reliance on current bioclimatic dimensions leaves open the possibility that species tolerances are underestimated because present-day climate represents only a subset of the species niche. This limitation becomes important when extrapolating species distributions into the future when novel climate conditions and new disturbance synergies are projected [5].

White or five-needled pines are considered keystone species of the subalpine environment, providing a food source for bears (*Ursus arctos* and *Ursus americanus*), red squirrel (*Tamiasciurus hudsonicus*), and Clark's nutcracker (*Nucifraga columbiana*) and playing an important role in stabilizing soil, moderating snowmelt and runoff, and facilitating establishment of other species [6–7]. In northwestern North America, there are two species of white pines: whitebark pine (*Pinus albicaulis*) and limber pine (*Pinus flexilis*). Whitebark pine is a native conifer that occurs at high elevations in the Sierra Nevada, Cascade Range, Pacific Coast Ranges and northern Rocky Mountains from Wyoming to the Continental Ranges of Canada. Limber pine grows under xeric conditions at lower and upper treeline in the mountains and foothills of the western U.S. and southern Canadian Rockies. Its distribution partially overlaps with that of whitebark pine in the northern Rocky Mountains, and the trees are difficult to distinguish unless cones are present. In the past two decades, white pines have experienced a notable decline within the northern Rocky Mountains that has been attributed to fire exclusion [8] and infestation from native mountain pine beetle (*Dendroctonus ponderosae*) and nonnative white pine blister rust (*Cronartium ribicola*) [9–10]. High mortality rates of whitebark pine in subalpine forests, in particular, have led to the U.S. Fish & Wildlife Service to warrant its protection under the Endangered Species Act.

Most niche models emphasize the susceptibility of white pines to current and future climate change, particularly their sensitivity to changes in summer temperature [11–12], whereas only a few models consider the importance of precipitation, biotic interactions or disturbance on its distribution and population dynamics [13–14]. According to temperature-based projections, for example, areas of suitable climate for whitebark pine, as an upper treeline species, are expected to be lost over much of the northern Rocky Mountains in the next 50 years as a result of rising summer temperatures and decreased effective moisture. In order to broaden the context for assessing its vulnerability at present and in the future, we examine the climate conditions, disturbance regimes and biotic associations under which white pines have persisted in the past. Our focus is the Greater Yellowstone region, a mountainous area (ca. 80,000 km^2^) that includes Yellowstone National Park and adjacent ecosystems. In specific, we reconstructed 15,000 years of vegetation and fire history by analyzing a network of pollen and charcoal records from low- to high-elevations sites (1598–3134 m) and compared these data with independent paleoenvironmental proxies. These radiocarbon-dated records reliably span the last 15,000 years, beginning with late-Pleistocene ice recession and extending to the present day, and provide a suite of climate and biotic scenarios that have no analog in the modern landscape.

## Methods

### Pollen and charcoal data

**Fig 1:**
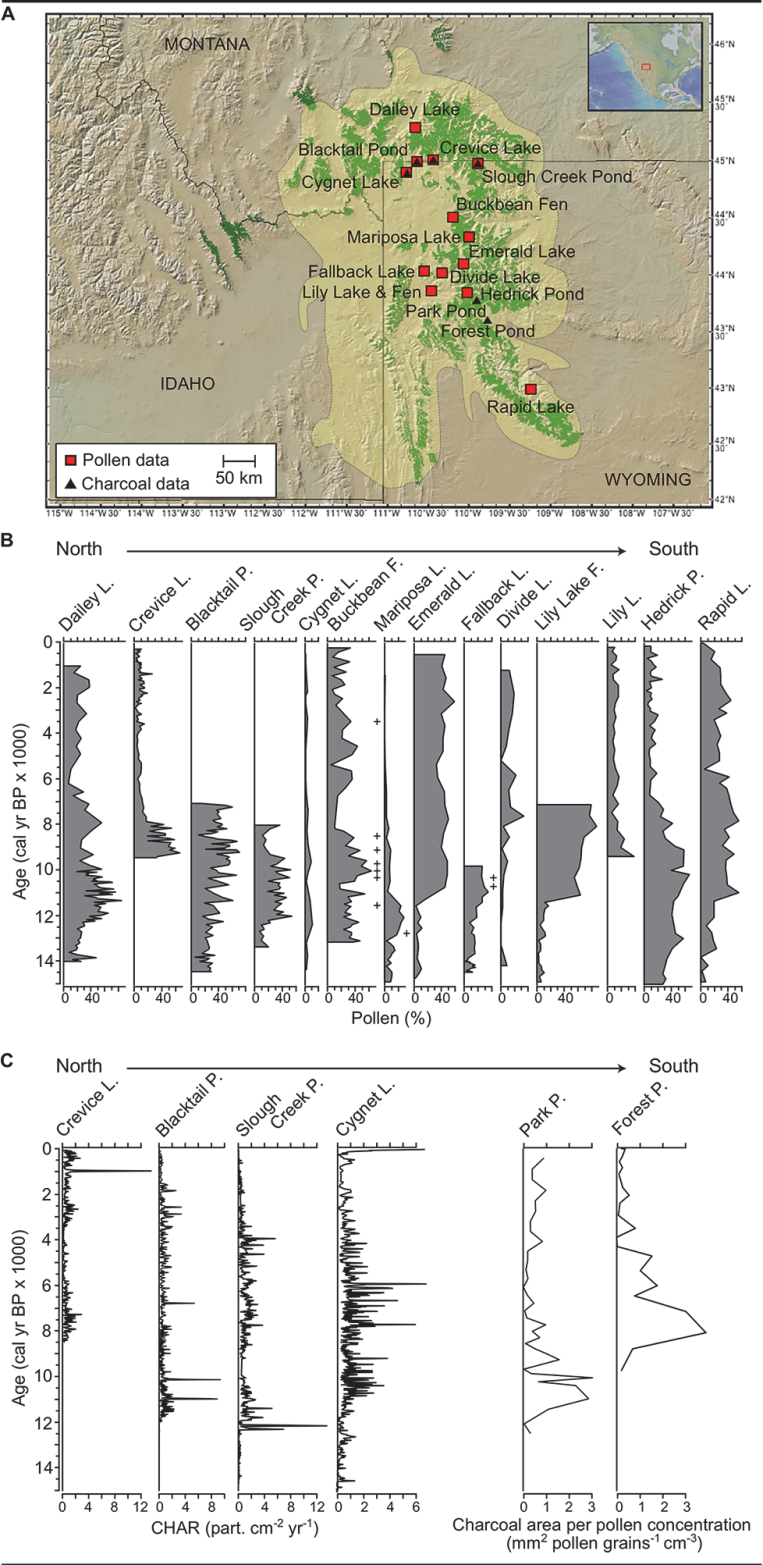
(A) Location of pollen and charcoal records in the Greater Yellowstone region (brown shade) and modern distribution of whitebark pine (green shade) (www.geomapapp.org) [15]. Postglacial trends in (B) *Pinus* subgenus *Strobus* pollen percentages, and (C) charcoal abundance at the study sites. The presence of macrofossils of *Pinus* subgenus *Strobus* is shown with ‘+’.

The vegetation and fire history reconstruction draws on 16 published radiocarbon-dated paleoenvironmental records from lake sediments at low to high elevations (1598–3134 m) and from the northern to southwestern extent of the Greater Yellowstone region (45.22–42.73° N, Fig 1, Table 1). All records are publicly available in the USGS North Central Paleoenvironmental Database (www.nccscpaleoenvironmentaldatabase.com).

**Table 1:** Study sites.

Site	Position	Elev. (m)	Modern vegetation	Data type
Blacktail Pond	44.96; -110.60	2012	Steppe-parkland	Pollen [16–17] & charcoal [17]
Buckbean Fen	44.29; -110.26	2362	*P*. *contorta—Picea*/*Abies*/*P*. *albicaulis* forest	Pollen [18]
Crevice Lake	45.00; -110.78	1684	Steppe-parkland/*Pseudotsuga*	Pollen & charcoal [19]
Cygnet Lake Fen	44.65; -110.60	2530	*P*. *contorta* forest	Pollen [20] & charcoal [21]
Dailey Lake	45.27; -110.82	1598	Steppe-parkland	Pollen [22]
Divide Lake	43.93: -110.23	2628	*Picea*/*Abies*/*P*. *albicaulis* forest	Pollen [20]
Emerald Lake	44.07; -110.30	2634	*Picea*/*Abies*/*P*. *albicaulis* forest	Pollen [20]
Fallback Lake	43.97; -110.43	2597	*Picea*/*Abies*/*P*. *albicaulis* forest	Pollen [20]
Forest Pond	43.37; -109.94	2797	*Picea*/*Abies*/*P*. *albicaulis* forest	Charcoal [23]
Hedrick Pond	43.75; -110.60	2073	Steppe-parkland	Pollen [20]
Lily Lake	43.77; -110.32	2469	*P*. *contorta—Picea*/*Abies*/*P*. *albicaulis* forest	Pollen [20]
Lily Lake Fen	43.77; -110.32	2469	*P*. *contorta—Picea*/*Abies*/*P*. *albicaulis* forest	Pollen [20]
Mariposa Lake	44.15; -110.23	2730	*P*. *contorta—Picea*/*Abies*/*P*. *albicaulis* forest	Pollen [20]
Park Pond	43.47; -109.96	2705	*Picea*/*Abies*/*P*. *albicaulis* forest	Charcoal [23]
Rapid Lake	42.73; -109.19	3134	Alpine meadow and tundra	Pollen [20]
Slough Creek Pond	44.93; -110.35	1884	Steppe-parkland	Pollen [24] & charcoal [21]

Changes in species abundance through time are inferred from temporal shifts in pollen percentages [25]. Specifically, our analysis focused on changes in the relative percentage of *Pinus* subgenus *Strobus* pollen, which is attributed to whitebark pine and/or limber pine in the Greater Yellowstone region. At present, limber pine is confined to low to middle elevations (less than 1900 m) and rocky exposures, while whitebark pine dominates at upper treeline (~2900 m elevation).

*Pinus* subgenus *Strobus* has distinctive pollen morphology from that of the subgenus *Pinus*, including lodgepole pine (*P*. *contorta*) and ponderosa pine (*P*. *ponderosa*). Changes in *Pinus* subgenus *Pinus* pollen percentages are ascribed to lodgepole pine based on the near-absence of ponderosa pine in the Greater Yellowstone region. Ponderosa pine does not grow at any of the study sites at present nor is there independent paleobotanical evidence to suggest its occurrence in the past 15,000 years.

Information on conifer history is obtained from stratigraphic pollen records [15–21] and inferred from the relationship between modern pollen rain and present-day vegetation. The relationship is influenced by (1) species-specific pollen productivity; (2) the aerodynamical properties of pollen grains, which affect their transport distance; (3) local topography, size, and microclimate of the watershed; and (4) the characteristics of the lake itself [26]. In mountainous regions with steep elevational gradients, significant amounts of pollen can be transported upslope thus overrepresenting the contribution of lower-elevation conifer taxa in pollen samples from high-elevation sites [27–29]. Particularly in these settings, the relationship between vegetation abundance and pollen percentages is non-linear (although always positive) and reconstructing local-scale vegetation history is challenging [30–31].

According to Prentice’s model of pollen source area [32], 62–63% of the *Pinus* pollen in a given sample from a medium-sized watershed is likely to have originated from within a 30 km radius of the lake. In an examination of modern pollen data from the western U.S., Minckley et al. found that *Pinus* pollen occurred at low-to-moderate percentages, with a median of 30% in alpine tundra and 25% in sagebrush steppe, and that high *Pinus* pollen abundance (>43%) was indicative of pine-dominated vegetation types [33]. In the Greater Yellowstone region, where topographic and vegetation gradients are relative gentle, modern pollen assemblages show high fidelity with elevationally-arrayed vegetation zones, enabling separation of steppe, middle-elevation forest, subalpine forest, and alpine communities. *Pinus* subgenus *Strobus*-type pollen in this region is most abundant in modern pollen samples from subalpine forests where *Pinus albicaulis* is present in large numbers [18, 20, 34]. However, even in the Greater Yellowstone region, conifer pollen percentages lack the spatial resolution to confirm local taxon presence without supporting paleobotanical information, such as plant macrofossils. Due to these limitations, we do not attempt to reconstruct watershed-scale population dynamics nor infer the local dynamics of white pines based on pollen abundance; instead our focus is on regional trends in the abundance of white pines. It is important to note that our reconstructions are of a qualitative nature and a linear relationship between pollen data and vegetation abundance is not assumed.

Past fire activity is inferred from trends in the accumulation of charcoal particles in lake-sediment cores from a network of six records from the Greater Yellowstone region. At Blacktail Pond [17], Crevice Lake [19], Slough Creek Pond and Cygnet Lake [21], trends in biomass burning were reconstructed from macroscopic charcoal accumulation rates (particles>250 μm diameter cm^-2^ yr^-1^) calculated from contiguous samples. These data have been shown to correlate well with biomass burning within a <30 km radius of the study site [35–36]. A different procedure was employed at Park Pond and Forest Pond [23], where microscopic charcoal particles (<100 μm diameter) on pollen slides were quantified as charcoal area per pollen concentration (mm^2^ pollen grains^-1^ cm^-3^). In order to reduce the influence of changes in sedimentation, non-influx data were converted to charcoal accumulation rates. Following Marlon et al. [37], differences in particle size, laboratory techniques and analytical approach were accounted for by standardizing all the records and expressing the data as anomalies with respect to the Holocene mean scaled by the standard deviation of each record.

### Time series analysis

Approximately 15,000 years of data, encompassing 922 pollen samples and 2399 charcoal samples were used to estimate regional trends in vegetation and fire activity with Generalized Additive Models (GAM, [38]; Table 2 and S1 Table). The data were constrained by 78 radiocarbon dates and 5 tephra layers of known age. Trends detected by the GAMs overcome the limitations of compounded single-site interpretations and, unlike other methods employed in data synthesis, do not require data interpolation. Additionally, GAMs allow specification of the distribution of the response variable and the link function and do not assume linearity [39]. Model selection was performed through comparison of their Akaike’s Information Criterion values (AIC [40]) calculated as:

AIC=2p-2ln(L) (1)

where p is the number or parameters in the model and L is the maximized value of the likelihood function of the model.

**Table 2:** Models employed in the reconstruction of regional trends in vegetation and fire in the Greater Yellowstone region.

Model		Family	ΔAIC	Deviance explained (%)
Pollen (%)				
*Abies*_i_ = α + f(Time_i_)_40_ + Site_i_ + ε_i_	(2)	Negative binomial	5.9	45.5
*Juniperus*_i_ = α + f(Time_i_)_10_ + Site_i_ + ε_i_	(3)	Negative binomial	248.0	46.0
*Picea*_i_ = α + f(Time_i_)_36_ + Site_i_ + ε_i_	(4)	Negative binomial	26.70	61.1
*P*. *Strobus*_i_ *=* α + f(Time_i_)_20_ + Site_i_ + ε_i_	(5)	Negative binomial	136.0	58.6
*P*. *Pinus*_i_ = α + f(Time_i_)_28_ + Site_i_ + ε_i_	(6)	Gaussian	151.5	75.5
*Pseudotsuga*_i_ = α + f(Time_i_)_22_ + Site_i_ + ε_i_	(7)	Negative binomial	123.0	63.4
Charcoal_i_ = α + f(Time_i_)_80_ + ε_i_	(8)	Gaussian	18.0	27.5

where pollen and charcoal data have been modeled as smoothing functions of the concatenated time data of all sites [f(Time_i_) = Time_ij_); with n = total number of covariates], the nominal variable Site_i_, an intercept α and the i^th^ residual error ε_i_.

We considered models within two AIC units to have equal predictive ability [41] and preferred those with the lowest AIC values (Table 2). Founded in information theory, model selection based on AIC reduction attempts to minimize information loss (i.e., increase goodness of fit) while penalizing complexity (i.e., overfitting).

### Spatial-temporal dynamics of white pines

Temporal changes in the abundance of white pines across an elevation gradient were reconstructed by interpolating *Pinus* subgenus *Strobus* pollen percentages onto a grid defined by time and elevation. In order to account for differences in the temporal resolutions of the records, we implemented Akima’s bilinear interpolation for irregularly spaced data [42]. All calculations and figures were made using R-programming language version 3.0.2 [43].

## Results and Discussion

**Fig 2:**
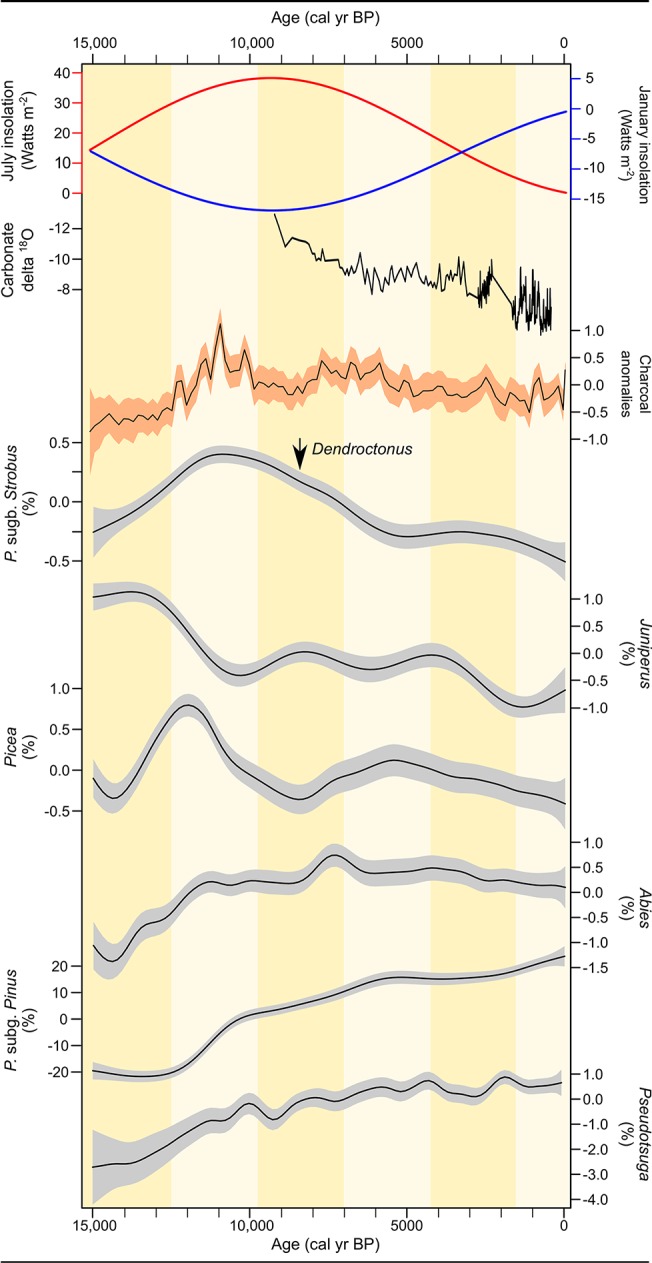
Environmental and conifer history in the Greater Yellowstone region over the last 15,000 years, including trends in January (blue) and July (red) insolation anomalies [44], snowpack dynamics inferred from δ^18^O variations at Crevice Lake [19] (note that the y-axis is reversed), fire activity (CHAR) and pollen abundance (%), and the first recorded presence of mountain pine beetle (*Dendroctonus* spp.) [46]. Carbonate δ^18^O variations at Crevice Lake are interpreted as changes in spring snowmelt that affect the isotopic composition of the Yellowstone River. Low (more negative) δ^18^O values correspond with more humid winters [19]. Pollen and charcoal regional trends are estimated by GAMs applied to the charcoal influx pollen percent and data. Standard errors are shown in gray.

Changes in the abundance of pollen taxa through time reflect long-term shifts in vegetation that are likely associated with large-scale variations in climate and their interaction with local environmental conditions (Fig 2). Prior to 13,000 cal yr BP (cal yr BP = calibrated radiocarbon years before AD 1950), the Greater Yellowstone region was characterized by sparsely vegetated deglacial landscapes. At the end of the Pleistocene and into the early Holocene (ca. 12,000 to 6000 cal yr BP), amplification of the seasonal cycle of insolation resulted in summer radiation values that were 8% higher than present and winter values that were 10% lower at lat 45°N [44]. Increased seasonality and associated changes in atmospheric circulation led to higher summer temperatures (~3°C above present, i.e., mean summer temperature over the 1998–2000 AD period), cold winters (2°C below present), and lower-than-present summer moisture (~0.5 mm day^-1^ below present) [45]. With the initial rise in postglacial growing-season temperatures, Engelmann spruce (*Picea engelmannii*; 13,500–11,000 cal yr BP), subalpine fir (*Abies lasiocarpa*; after 12,500 cal yr BP), and white pines (after 12,500 cal yr BP) established in the region to form open parkland and then closed subalpine forest (Fig 2). The pollen data suggest greatest abundance of whitebark pine and/or limber pine between 12,000 and 7500 cal yr BP at most sites, and a steady decline in abundance thereafter, tracking the decrease in summer insolation and rise in winter insolation. The presence of *Pinus cf*. *P*. *albicaulis* needles in late-glacial/early-Holocene sediments at some sites confirmed the early local presence of this taxon (Fig 1) [20]. Lodgepole pine steadily increased in abundance after ~11,000 cal yr BP and became the dominant conifer at middle elevations and on rhyolitic volcanic substrates [21]. Douglas-fir (*Pseudotsuga menziesii*) expanded at low and middle elevations after 7000 cal yr BP (Fig 2).

**Fig 3:**
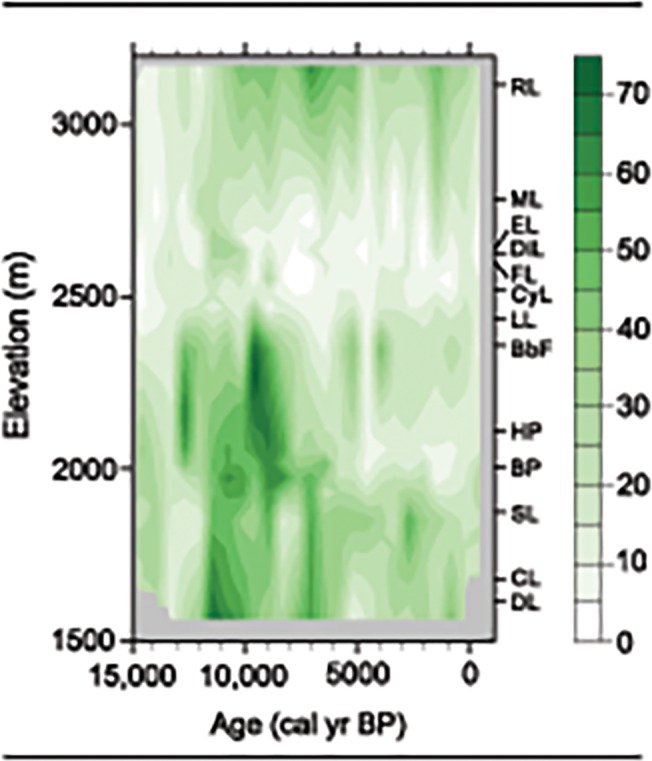
Spatial-temporal dynamics of *Pinus* subgenus *Strobus* pollen, attributed to whitebark pine and limber pine. Darker shades of green show increasing pollen representation on a grid defined by elevation and time. The elevation of the sites employed in the analysis is shown on the right (RL: Rapid Lake; ML: Mariposa Lake; EL: Emerald Lake; DiL: Divide Lake; FL: Fallback Lake; CyL: Cygnet Lake; LL: Lily Lake and fen; BbF: Buckbean Fen; HP: Hedrick Pond; BP: Blacktail Pond; SL: Slough Creek Pond; CL: Crevice Lake; DL: Dailey Lake).

Interpolated pollen data of *Pinus* subgenus *Strobus* from the 14 records highlight temporal and elevational changes in white pine abundance and distribution over the last 15,000 cal yrs (Fig 3). The full-glacial distribution of whitebark and limber pine in the region is unknown in the absence of sites from nonglaciated areas, but *Pinus* subgenus *Strobus* pollen was present early in the deglacial history when tundra and subalpine parkland were widespread. Initial expansion occurred at middle elevations (2000–2400 m) at 13,000 cal yr BP; and between 12,000 and 9000 cal yr BP, whitebark and/or limber pine were well represented at most elevations. The abundance of other subalpine conifers (e.g., Engelmann spruce and subalpine fir) in the pollen record prior to 9000 cal yr BP (Fig 2) suggests that whitebark pine was the likely contributor of the early *Pinus* subgenus *Strobus* pollen, but the presence of limber pine cannot be ruled out. After 9000 cal yr BP, and especially after 4000 cal yr BP, the interpolated pollen patterns show distinct high-elevation and a low-elevation components in the *Pinus* subgenus *Strobus* record, which we attribute the restriction of whitebark pine to upper treeline forests and limber pine to low elevation forests.

Pollen data suggest that white pine populations in the Greater Yellowstone region had sufficient phenotypic plasticity/genetic variability to survive large-magnitude variations in postglacial climate. The observation that *Pinus* subgenus *Strobus* pollen was most abundant during the interval of highest summer insolation (ca. 12,000–7500 cal yr BP), when summers were warmer and effectively drier than present, implies that elevated summer temperatures and drought are not a primary constraint on the distribution of white pines. Conversely, regional abundance of whitebark and/or limber pine declined in the middle and late Holocene, when decreasing summer and rising winter insolation led to cooler summer temperatures and warmer winters than before.

Independent information on winter precipitation trends over the last 9800 cal yr comes from carbonate δ^18^O data at Crevice Lake, a middle elevation site where the paleohydrology is influenced by Yellowstone River discharge and snowmelt from higher elevations [19]. Progressive oxygen isotope enrichment of endogenic carbonates at Crevice Lake after 8000 cal yr BP and particularly after 3000 cal yr BP indicates a trend of decreasing winter precipitation in the middle and late Holocene (Fig 2). Winter precipitation is critical in shaping summer soil moisture in the region, and this long-term trend towards less snowpack and seasonal drought, at least at low and middle elevations, likely contributed to the restriction of whitebark pine and other subalpine species to high elevations towards present day. The white pine-climate relationship evident from the paleorecord indicates a higher tolerance for warmer drier summers and larger susceptibility to warmer and drier winter conditions than its present distribution would imply.

In addition to climate influences, the abundance of white pines at present is less than in the past as a result of long-term changes in disturbance regimes and biotic interactions. Ecological data suggest that, in the absence of disturbance, whitebark pine and limber pine are replaced by more shade-tolerant species, such as Engelmann spruce and subalpine fir at high elevations [47] and Douglas-fir at low elevations [48]. Charcoal records, which describe regional fire activity, indicate highest levels of fire between 12,500 and 10,000 cal yr BP, when whitebark pine and possibly limber pine expanded at the expense of Engelmann spruce and subalpine fir (Fig 2). This shift in dominance would have been favored by the greater resilience of white pines to fire, in as much as their seeds are widely dispersed by Clark’s nutcrackers after disturbance, and seedlings establish quickly on exposed burned sites [49, 50]. In contrast, Engelmann spruce and subalpine fir seeds rely solely on wind dispersal, and seedlings require moist soil and shade to establish, and are less tolerant of exposed sites and drought than those of either whitebark pine or limber pine. Paleoecological reconstructions thus accord with modern ecological observations by showing that the competitive exclusion of whitebark pine and limber pine by shade-tolerant conifers is most significant under conditions of low disturbance [51]. This hypothesis being true, the increase in Engelmann spruce and subalpine fir over whitebark pine in high-elevation regions observed in recent decades may be partly explained by a reduction of fire resulting from management practices [52].

The biogeographic distribution of whitebark pine is also influenced by competition with shade-intolerant lodgepole pine. Despite its dominance at upper treeline, small individuals of whitebark pine compete in the understory throughout the subalpine forest zone, including where lodgepole pine is dominant. Lodgepole pine regenerates quickly after disturbance and grows faster than whitebark pine [53]; where they co-occur, lodgepole pine creates a closed-canopy forest that suppresses whitebark pine development. Pollen data suggest that this strong interaction developed after 11,000 cal yr BP when lodgepole pine expanded its range at middle elevations, and by 7500 cal yr BP, whitebark pine was restricted to high-elevation sites (Fig 3). In the absence of lodgepole pine, it is likely that whitebark pine would be more abundant in subalpine forests than it is, despite decreased snowpack, low fire activity, and insect outbreaks. At low and middle elevations, the expansion of Douglas-fir and lodgepole pine in the middle and late Holocene (Fig 2) may have restricted the occurrence of limber pine in a similar manner.

The paleoecological record has less to say about other biotic interactions that may have influenced the range of white pines in the Greater Yellowstone region. Remains of mountain pine beetle recovered from lake sediments dating to the middle and late Holocene indicate that *Dendroctonous* sp. has been present in the Northern Rocky Mountains for at least 8000 cal yr BP [46]. Although population dynamics cannot be inferred from these data, recent outbreaks are considered unusual in that beetles typically display univoltine rather than semivoltine reproduction. Therefore, instead of requiring two years to regenerate, mountain pine beetles are now able to complete their life cycle in a single year, which greatly increases the probability and magnitude of the current outbreaks [54]. The shortened life cycle is attributed to increased winter temperatures, which allow survival of the larvae through the cold season. In this respect, the present outbreak may arise from unprecedented climate conditions (i.e., highest winter temperatures in the Holocene), and the vulnerability of white pines to insect infestations will likely be intensified by projected warmer winters.

## Conclusions

Our study offers a new approach for assessing present and future vulnerability of white pines to environmental change. By analyzing past responses at a regional scale, we overcome some of the limitations of interpreting species histories site by site, especially in complex mountain environments where local site variability can strongly influence species sensitivity to climate change. Pollen data indicate that the long-term susceptibility of the region’s conifers to past climate variations, fire conditions, and biotic interactions differs from our understanding based on associations between modern climate and tree abundance. Such differences highlight the shortcomings of extrapolating bioclimatic data beyond observed correlations and suggest that (i) relationships between present-day species distributions and modern climate only define a fraction of their niche and may underestimate species capacity to respond to climate variability; (ii) modern relationships based on mature individuals may not capture the conditions required for seedling establishment and growth across the species range; and (iii) the importance of biotic interactions (e.g., competition, predation) is underestimated in many ecological niche studies because of co-linearity between climate and other species abundance.

Despite these limitations, estimating future biogeographic ranges is key to natural resource planning and conservation. Integration of multiple disciplines in creating robust assessments of climate change offers a powerful tool for management in the face of an uncertain future [5]. Paleoenvironmental data contribute to these efforts by providing information on vegetation responses to a broader array of environmental conditions than can be observed at present. Past climate-vegetation relationships indicate that white pines are more vulnerable to projected warmer drier winters than to summer conditions or fire activity. The added effects of increased mountain pine beetle and blister rust outbreaks, in combination with the superior ability of lodgepole pine to respond to changes in disturbance regimes, stand out as a suite of critical factors with no historical precedence [55]. Current conservation strategies that reduce competition with other conifers, support natural fire regimes, and utilize blister-rust resistant strains align well with our understanding of the long-term controls that maintain white pines as components of northern Rocky Mountain forests [56].

## Supporting Information

S1 Table Estimated intercepts and standard errors for the models applied to the pollen data. (DOCX)
