# Supplementary material for: Complex Response of White Pines to Past Environmental Variability Increases Understanding of Future Vulnerability
Source: PLoS One. 2015 Apr 17;10(4):e0124439. doi: 10.1371/journal.pone.0124439 (PMC4401641; doi:10.1371/journal.pone.0124439)
Supplement: S1 Table — (DOCX) [file pone.0124439.s001.docx]

| Site | *Abies* | *Juniperus* | *Picea* | *P. Strobus* | *P. Pinus* | *Pseudotsuga* | Charcoal |
| --- | --- | --- | --- | --- | --- | --- | --- |
| Blacktail Pond | -0.4 +/-0.1 | -0.7+/-0.1 | 0.9 +/-0.1 | 3.3+/-0.0 | 30.8+/-1.1 | -0.5+/-0.1 | 0+/-0.01 |
| Buckbean Fen | 0.2 +/-0.2 | 1.1+/-0.2 | -0.6 +/-0.1 | 0.0+/-0.1 | 5.1+/-1.8 | -1.8+/-0.3 | NA |
| Crevice Lake | 0.1 +/-0.1 | 2.0+/-0.5 | NA | 0.9 +/-0.3 | 0.4+/-1.6 | 0.9+/-0.1 | 0+/-0.01 |
| Cygnet Lake | -0.1 +/-0.2 | 0.4+/-0.2 | -1.0 +/-0.2 | -2.1+/-0.1 | 2.3+/-2.3 | -1.9+/-0.4 | 0+/-0.01 |
| Dailey Lake | 1.0 +/-0.1 | 0.9+/-0.2 | -0.2 +/-0.2 | 0.1+/-0.1 | -6.4+/-1.7 | 0.0+/-0.2 | NA |
| Divide Lake | 1.8 +/-0.2 | -0.5+/-0.4 | 1.9 +/-0.1 | -0.8+/-0.1 | 9.0+/-2.6 | -0.2+/-0.3 | NA |
| Emerald Lake | 2.1 +/-0.1 | 0.5+/-0.2 | 1.6 +/-0.1 | -0.8+/-0.1 | 1.4+/-2.2 | -1.1+/-0.3 | NA |
| Fallback Lake | 1.9 +/-0.2 | 0.5+/-0.2 | 0.8 +/-0.1 | -0.8+/-0.1 | 5.2+/-2.4 | -0.7+/-0.5 | NA |
| Forest Pond | NA | NA | NA | NA | NA | NA | 0+/-0.01 |
| Hedrick Pond | 0.6 +/-0.2 | 0.3+/-0.2 | 0.6 +/-0.1 | -0.2+/-0.1 | 3.6+/-1.8 | -0.2+/-0.2 | NA |
| Lily Lake & Fen | 0.3 +/-0.2 | 0.8+/-0.2 | 0.6 +/-0.1 | -0.5+/-0.1 | 13.6+/-2.3 | 0.2+/-0.3 | NA |
| Mariposa Lake | 1.3 +/-0.2 | 0.2+/-0.2 | 0.9 +/-0.1 | -1.2+/-0.1 | 19.6+/-2.0 | -2.4+/-0.7 | NA |
| Park Pond | NA | NA | NA | NA | NA | NA | 0+/-0.01 |
| Rapid Lake | 0.9 +/-0.2 | 1.2+/-0.2 | 1.1 +/-0.1 | 0.1+/-0.1 | NA | -2.0+/-0.4 | NA |
| Slough Creek Pond | 0.5 +/-0.1 | 1.8+/-0.2 | 0.7 +/-0.1 | 0.3+/-0.1 | 2.2+/-2.1 | -1.5+/-0.4 | 0+/-0.01 |

**Table S1.** Estimated intercepts and standard errors for the models applied to the pollen data.
